# 2,6-Bis(1*H*-benzimidazol­-2-yl)pyridine methanol trisolvate

**DOI:** 10.1107/S1600536809012574

**Published:** 2009-04-10

**Authors:** Ying Chen, Jixi Guo, Xingcai Huang, Ruirui Yun, Huilu Wu

**Affiliations:** a School of Chemical and Biological Engineering, Lanzhou Jiaotong University, Lanzhou 730070, People’s Republic of China; b Institute of Applied Chemistry, Xinjiang University, Urumqi 830046, Xinjiang, People’s Republic of China

## Abstract

In the title compound, C_19_H_13_N_5_·3CH_4_O, the 2,6-bis­(2-benzimidazol­yl)pyridine mol­ecule is essentially planar with an r.m.s. deviation for all non-H atoms of 0.185 Å. The crystal structure is stabilized by inter­molecular O—H⋯O, O—H⋯N and N—H⋯O hydrogen bonds and weak π⋯π stacking inter­actions with centroid–centroid distances of 3.6675 (16) and 3.6891 (15) Å. The atoms of one of the methanol solvent molecules are disordered over two sites with refined occupancies of 0.606(8) and 0.394(8).

## Related literature

For the crystal structures of the mono- and sesquihydrate analogs of 2,6-bis­(2-benzimidazol­yl)pyridine, see: Freire *et al.* (2003 ▶). For the synthesis of 2,6-bis­(*2*-benzimidazol­yl)pyridine, see: Addison & Burke (1981 ▶).
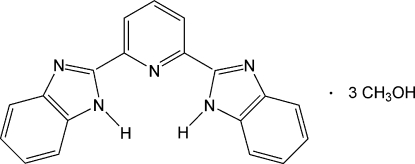


## Experimental

### 

#### Crystal data

C_19_H_13_N_5_·3CH_4_O
*M* *_r_* = 407.47
Monoclinic, 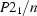

*a* = 11.2686 (9) Å
*b* = 15.0928 (13) Å
*c* = 13.0679 (11) Å
β = 107.391 (2)°
*V* = 2120.9 (3) Å^3^
*Z* = 4
Mo *K*α radiation
μ = 0.09 mm^−1^
*T* = 153 K
0.18 × 0.14 × 0.11 mm

#### Data collection

Rigaku R-AXIS Spider diffractometer
Absorption correction: multi-scan (Higashi, 1995 ▶) *T*_min_ = 0.984, *T*_max_ = 0.990
17035 measured reflections
3945 independent reflections
2527 reflections with *I* > 2σ(*I*)
*R*_int_ = 0.071

#### Refinement

*R*[*F* ^2^ > 2σ(*F* ^2^)] = 0.077
*wR*(*F* ^2^) = 0.236
*S* = 1.04
3945 reflections
307 parameters
2 restraints
H atoms treated by a mixture of independent and constrained refinement
Δρ_max_ = 0.39 e Å^−3^
Δρ_min_ = −0.40 e Å^−3^

### 

Data collection: *RAPID-AUTO* (Rigaku/MSC 2004 ▶); cell refinement: *RAPID-AUTO*; data reduction: *RAPID-AUTO*; program(s) used to solve structure: *SHELXS97* (Sheldrick, 2008 ▶); program(s) used to refine structure: *SHELXL97* (Sheldrick, 2008 ▶); molecular graphics: *SHELXTL* (Sheldrick, 2008 ▶) and *PLATON* (Spek, 2009 ▶); software used to prepare material for publication: *SHELXTL*.

## Supplementary Material

Crystal structure: contains datablocks global, I. DOI: 10.1107/S1600536809012574/lh2799sup1.cif

Structure factors: contains datablocks I. DOI: 10.1107/S1600536809012574/lh2799Isup2.hkl

Additional supplementary materials:  crystallographic information; 3D view; checkCIF report

## Figures and Tables

**Table 1:** Hydrogen-bond geometry (Å, °)

*D*—H⋯*A*	*D*—H	H⋯*A*	*D*⋯*A*	*D*—H⋯*A*
O1—H1⋯O2^i^	0.84	1.83	2.670 (3)	176
O2—H2⋯N4	0.84	1.91	2.741 (3)	168
N1—H1*N*⋯O1	0.866 (10)	2.069 (12)	2.927 (3)	171 (3)
N3—H3*N*⋯O1	0.863 (10)	2.069 (12)	2.925 (3)	171 (4)

Symmetry code: (i) 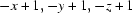.

**Fig. 1.**
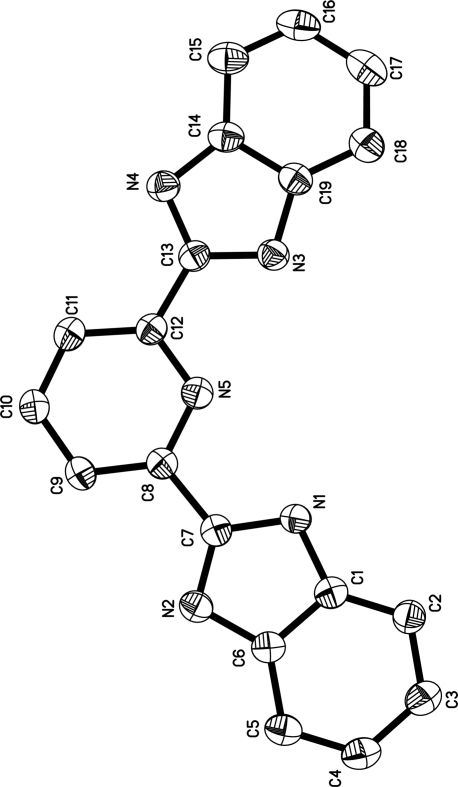
The molecular structure of the title compound. Displacement ellipsoids are drawn at the 30% probability level. H atoms and solvent molecules have been omitted for clarity.

**Fig. 2.**
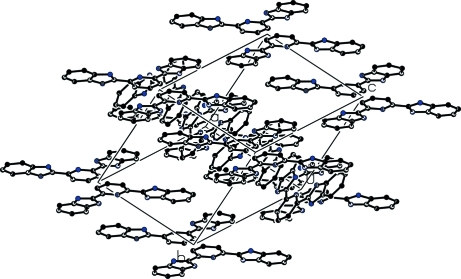
Part of the crystal structure showing weak π···π stacking interactions. The solvent molecules are not shown

## supplementary crystallographic information

### Comment

The synthesis of 2,6-bis(2-benzimidazolyl)pyridine has been reported in the literature (Addison & Burke 1981) and the crystal structures of the mono and sesqihydrates of this compound have been determined (Freire *et al.*, 2003). During our studies of benzimidazole complexes involving a recrystallization of 2,6-bis(2-benzimidazolyl)pyridine from methanol we unexpectedly form the trimethanol solvate (I).

The molecular structure of the 2,6-bis(2-benzimidazolyl)pyridine molecule is shown in Fig. 1. The molecule is essentially planar with a rms deviation of all non-hydrogen fitted atoms = 0.185. The crystal structure is stabilized by intermolecular hydrogen bonds (see Table 1) and weak π···π stacking interactions (Fig. 2) with, centroid to centroid distances of 3.6675 (16) and 3.6891 (15)Å, between pryridine rings and benzimidazole rings of inversion related molecules.

### Experimental

2,6-bis(2-benzimidazolyl)pyridine was prepared by the method of Addison & Burke (1981). After recrystallization from methanol, fine white needles were formed. The mother liquor was set aside for several days leading to the formation of crystals that were suitable for X-ray diffraction analysis.

### Refinement

All H atoms were found in difference electron maps and were subsequently refined in a riding-model approximation with C—H distances ranging from 0.95 to 0.98 Å and with *U*_iso_(H) = 1.2 *U*_eq_(C) for CH and *U*_iso_(H) = 1.2 *U*_eq_(O) for OH. H atoms bonded to N atoms were refined independently with isotropic displacement parameters. The atoms of one methanol solvent molecule is disordered over two sites with refined occupancies of 0.606 (8) and 0.394 (8).

### Crystal data

**Table:**

C_19_H_13_N_5_·3CH_4_O	*F*(000) = 864
*M**_r_* = 407.47	*D*_x_ = 1.276 Mg m^−^^3^
Monoclinic, *P*2_1_/*n*	Mo *K*α radiation, λ = 0.71073 Å
Hall symbol: -P 2yn	Cell parameters from 3945 reflections
*a* = 11.2686 (9) Å	θ = 3.2–25.5°
*b* = 15.0928 (13) Å	µ = 0.09 mm^−^^1^
*c* = 13.0679 (11) Å	*T* = 153 K
β = 107.391 (2)°	Block, colorless
*V* = 2120.9 (3) Å^3^	0.18 × 0.14 × 0.11 mm
*Z* = 4	

### Data collection

**Table:**

Rigaku R-AXIS Spider diffractometer	3945 independent reflections
Radiation source: fine-focus sealed tube	2527 reflections with *I* > 2σ(*I*)
graphite	*R*_int_ = 0.071
φ and ω scans	θ_max_ = 25.5°, θ_min_ = 3.2°
Absorption correction: multi-scan (Higashi, 1995)	*h* = −13→11
*T*_min_ = 0.984, *T*_max_ = 0.990	*k* = −18→18
17035 measured reflections	*l* = −15→15

### Refinement

**Table:**

Refinement on *F*^2^	Secondary atom site location: difference Fourier map
Least-squares matrix: full	Hydrogen site location: inferred from neighbouring sites
*R*[*F*^2^ > 2σ(*F*^2^)] = 0.077	H atoms treated by a mixture of independent and constrained refinement
*wR*(*F*^2^) = 0.236	*w* = 1/[σ^2^(*F*_o_^2^) + (0.1505*P*)^2^] where *P* = (*F*_o_^2^ + 2*F*_c_^2^)/3
*S* = 1.04	(Δ/σ)_max_ = 0.002
3945 reflections	Δρ_max_ = 0.39 e Å^−^^3^
307 parameters	Δρ_min_ = −0.40 e Å^−^^3^
2 restraints	Extinction correction: *SHELXL*, Fc^*^=kFc[1+0.001xFc^2^λ^3^/sin(2θ)]^-1/4^
Primary atom site location: structure-invariant direct methods	Extinction coefficient: 0.040 (6)

### Special details

**Table:**

Geometry. All e.s.d.'s (except the e.s.d. in the dihedral angle between two l.s. planes) are estimated using the full covariance matrix. The cell e.s.d.'s are taken into account individually in the estimation of e.s.d.'s in distances, angles and torsion angles; correlations between e.s.d.'s in cell parameters are only used when they are defined by crystal symmetry. An approximate (isotropic) treatment of cell e.s.d.'s is used for estimating e.s.d.'s involving l.s. planes.
Refinement. Refinement of *F*^2^ against ALL reflections. The weighted *R*-factor *wR* and goodness of fit *S* are based on *F*^2^, conventional *R*-factors *R* are based on *F*, with *F* set to zero for negative *F*^2^. The threshold expression of *F*^2^ > σ(*F*^2^) is used only for calculating *R*-factors(gt) *etc*. and is not relevant to the choice of reflections for refinement. *R*-factors based on *F*^2^ are statistically about twice as large as those based on *F*, and *R*- factors based on ALL data will be even larger.

### Fractional atomic coordinates and isotropic or equivalent isotropic displacement parameters (Å^2^)

**Table:**

	*x*	*y*	*z*	*U*_iso_*/*U*_eq_	Occ. (<1)
O1	0.20534 (18)	0.61337 (13)	0.36351 (15)	0.0631 (6)	
H1	0.2509	0.6582	0.3816	0.076*	
O2	0.65988 (19)	0.23950 (14)	0.57774 (17)	0.0704 (6)	
H2	0.6009	0.2626	0.5299	0.084*	
N1	0.11042 (19)	0.58598 (15)	0.54657 (17)	0.0477 (6)	
N2	0.0754 (2)	0.53929 (15)	0.69788 (17)	0.0517 (6)	
N3	0.3694 (2)	0.46017 (15)	0.38227 (18)	0.0510 (6)	
N4	0.4825 (2)	0.33787 (15)	0.43304 (18)	0.0536 (6)	
N5	0.27120 (19)	0.45211 (14)	0.54781 (16)	0.0492 (6)	
C1	0.0280 (2)	0.64631 (18)	0.5671 (2)	0.0498 (7)	
C2	−0.0274 (2)	0.72243 (19)	0.5148 (2)	0.0558 (7)	
H2A	−0.0109	0.7432	0.4518	0.067*	
C3	−0.1076 (3)	0.7667 (2)	0.5589 (2)	0.0609 (8)	
H3	−0.1476	0.8191	0.5254	0.073*	
C4	−0.1313 (3)	0.7359 (2)	0.6519 (2)	0.0615 (8)	
H4	−0.1875	0.7680	0.6795	0.074*	
C5	−0.0761 (2)	0.6613 (2)	0.7043 (2)	0.0558 (7)	
H5	−0.0930	0.6411	0.7674	0.067*	
C6	0.0060 (2)	0.61602 (18)	0.6613 (2)	0.0500 (7)	
C7	0.1350 (2)	0.52423 (17)	0.6263 (2)	0.0479 (6)	
C8	0.2211 (2)	0.45155 (17)	0.62863 (19)	0.0467 (7)	
C9	0.2477 (2)	0.38735 (18)	0.7084 (2)	0.0513 (7)	
H9	0.2096	0.3888	0.7641	0.062*	
C10	0.3312 (2)	0.32126 (18)	0.7045 (2)	0.0536 (7)	
H10	0.3520	0.2767	0.7582	0.064*	
C11	0.3837 (2)	0.32049 (18)	0.6221 (2)	0.0529 (7)	
H11	0.4411	0.2755	0.6181	0.063*	
C12	0.3512 (2)	0.38721 (17)	0.5444 (2)	0.0471 (7)	
C13	0.4022 (2)	0.39322 (17)	0.4542 (2)	0.0480 (7)	
C14	0.5051 (2)	0.37188 (19)	0.3421 (2)	0.0539 (7)	
C15	0.5848 (3)	0.3422 (2)	0.2854 (2)	0.0656 (8)	
H15	0.6334	0.2901	0.3066	0.079*	
C16	0.5905 (3)	0.3906 (2)	0.1981 (3)	0.0695 (9)	
H16	0.6442	0.3717	0.1584	0.083*	
C17	0.5188 (3)	0.4674 (2)	0.1662 (2)	0.0713 (9)	
H17	0.5244	0.4990	0.1049	0.086*	
C18	0.4406 (3)	0.4980 (2)	0.2211 (2)	0.0616 (8)	
H18	0.3930	0.5504	0.1999	0.074*	
C19	0.4344 (2)	0.44882 (18)	0.3089 (2)	0.0531 (7)	
C20	0.1118 (3)	0.6305 (3)	0.2645 (3)	0.0789 (10)	
H20A	0.0453	0.6661	0.2780	0.095*	
H20B	0.0773	0.5742	0.2312	0.095*	
H20C	0.1484	0.6628	0.2163	0.095*	
C21	0.7174 (4)	0.1739 (3)	0.5314 (4)	0.0958 (12)	
H21A	0.7639	0.2025	0.4879	0.115*	
H21B	0.6536	0.1350	0.4858	0.115*	
H21C	0.7745	0.1389	0.5883	0.115*	
O3	0.1278 (5)	0.5658 (3)	0.9743 (4)	0.094 (2)	0.606 (8)
H3A	0.1412	0.5425	1.0350	0.112*	0.606 (8)
C22	0.2186 (8)	0.5366 (7)	0.9269 (9)	0.0521 (19)	0.606 (8)
H22A	0.2730	0.5862	0.9227	0.062*	0.606 (8)
H22B	0.1774	0.5142	0.8547	0.062*	0.606 (8)
H22C	0.2681	0.4892	0.9707	0.062*	0.606 (8)
O3'	0.0859 (9)	0.4916 (6)	0.8986 (6)	0.118 (4)	0.394 (8)
H3'	0.0325	0.5092	0.9272	0.141*	0.394 (8)
C22'	0.1816 (12)	0.5468 (13)	0.9232 (18)	0.078 (5)	0.394 (8)
H22D	0.1522	0.6072	0.9022	0.094*	0.394 (8)
H22E	0.2405	0.5293	0.8848	0.094*	0.394 (8)
H22F	0.2230	0.5449	1.0006	0.094*	0.394 (8)
H1N	0.144 (3)	0.589 (2)	0.4954 (17)	0.075 (10)*	
H3N	0.316 (3)	0.5018 (17)	0.380 (3)	0.095 (12)*	

### Atomic displacement parameters (Å^2^)

**Table:**

	*U*^11^	*U*^22^	*U*^33^	*U*^12^	*U*^13^	*U*^23^
O1	0.0689 (13)	0.0565 (13)	0.0638 (13)	0.0040 (9)	0.0197 (10)	0.0078 (9)
O2	0.0655 (13)	0.0665 (14)	0.0811 (15)	0.0113 (10)	0.0249 (11)	0.0109 (11)
N1	0.0473 (12)	0.0518 (13)	0.0463 (12)	0.0027 (10)	0.0175 (10)	0.0002 (10)
N2	0.0524 (12)	0.0558 (14)	0.0479 (12)	−0.0030 (10)	0.0164 (10)	−0.0030 (10)
N3	0.0535 (13)	0.0505 (14)	0.0516 (13)	−0.0013 (10)	0.0196 (10)	−0.0001 (10)
N4	0.0559 (13)	0.0514 (14)	0.0553 (13)	−0.0005 (10)	0.0194 (10)	−0.0041 (10)
N5	0.0515 (12)	0.0491 (13)	0.0443 (12)	−0.0018 (10)	0.0104 (10)	−0.0040 (9)
C1	0.0490 (14)	0.0486 (15)	0.0505 (14)	−0.0026 (12)	0.0130 (11)	−0.0065 (12)
C2	0.0579 (15)	0.0553 (17)	0.0534 (15)	0.0034 (13)	0.0154 (13)	0.0005 (13)
C3	0.0564 (16)	0.0600 (18)	0.0634 (17)	0.0060 (13)	0.0134 (14)	−0.0076 (14)
C4	0.0529 (15)	0.067 (2)	0.0655 (18)	0.0034 (14)	0.0188 (14)	−0.0138 (15)
C5	0.0523 (15)	0.0639 (19)	0.0538 (15)	−0.0056 (13)	0.0195 (12)	−0.0095 (13)
C6	0.0464 (13)	0.0524 (16)	0.0508 (14)	−0.0025 (12)	0.0140 (11)	−0.0058 (12)
C7	0.0495 (14)	0.0462 (15)	0.0470 (14)	−0.0030 (11)	0.0130 (11)	−0.0019 (11)
C8	0.0474 (14)	0.0469 (15)	0.0446 (13)	−0.0029 (11)	0.0119 (11)	−0.0032 (11)
C9	0.0551 (15)	0.0542 (17)	0.0440 (14)	−0.0031 (12)	0.0136 (12)	0.0031 (11)
C10	0.0587 (16)	0.0499 (16)	0.0518 (15)	0.0000 (12)	0.0159 (13)	0.0066 (12)
C11	0.0517 (15)	0.0486 (16)	0.0548 (15)	0.0031 (12)	0.0106 (12)	0.0008 (12)
C12	0.0474 (14)	0.0437 (15)	0.0480 (14)	−0.0022 (11)	0.0108 (11)	−0.0047 (11)
C13	0.0478 (14)	0.0440 (15)	0.0515 (14)	−0.0022 (11)	0.0140 (11)	−0.0038 (11)
C14	0.0536 (15)	0.0539 (17)	0.0570 (16)	−0.0096 (12)	0.0211 (13)	−0.0116 (13)
C15	0.0624 (17)	0.068 (2)	0.0706 (19)	−0.0118 (15)	0.0260 (15)	−0.0182 (16)
C16	0.0716 (19)	0.076 (2)	0.070 (2)	−0.0209 (17)	0.0358 (16)	−0.0247 (17)
C17	0.081 (2)	0.082 (2)	0.0552 (17)	−0.0275 (18)	0.0271 (16)	−0.0105 (16)
C18	0.0663 (17)	0.0607 (19)	0.0588 (16)	−0.0094 (14)	0.0202 (14)	−0.0025 (14)
C19	0.0548 (15)	0.0552 (17)	0.0486 (15)	−0.0087 (12)	0.0145 (12)	−0.0072 (12)
C20	0.073 (2)	0.091 (3)	0.070 (2)	−0.0099 (18)	0.0166 (17)	0.0182 (18)
C21	0.087 (2)	0.073 (3)	0.131 (3)	0.0229 (19)	0.037 (2)	0.005 (2)
O3	0.106 (4)	0.102 (4)	0.070 (3)	−0.012 (3)	0.021 (3)	0.001 (2)
C22	0.024 (4)	0.086 (5)	0.050 (3)	0.011 (3)	0.016 (4)	0.010 (3)
O3'	0.130 (8)	0.127 (7)	0.088 (5)	−0.044 (6)	0.020 (5)	0.001 (5)
C22'	0.021 (7)	0.147 (13)	0.070 (7)	0.039 (7)	0.019 (6)	0.000 (6)

### Geometric parameters (Å, °)

**Table:**

O1—C20	1.427 (3)	C10—H10	0.9500
O1—H1	0.8400	C11—C12	1.399 (4)
O2—C21	1.415 (4)	C11—H11	0.9500
O2—H2	0.8400	C12—C13	1.460 (4)
N1—C7	1.363 (3)	C14—C15	1.398 (4)
N1—C1	1.383 (3)	C14—C19	1.402 (4)
N1—H1N	0.866 (10)	C15—C16	1.372 (5)
N2—C7	1.324 (3)	C15—H15	0.9500
N2—C6	1.399 (3)	C16—C17	1.404 (5)
N3—C13	1.354 (3)	C16—H16	0.9500
N3—C19	1.381 (4)	C17—C18	1.372 (4)
N3—H3N	0.863 (10)	C17—H17	0.9500
N4—C13	1.321 (3)	C18—C19	1.385 (4)
N4—C14	1.386 (4)	C18—H18	0.9500
N5—C8	1.338 (3)	C20—H20A	0.9800
N5—C12	1.341 (3)	C20—H20B	0.9800
C1—C2	1.386 (4)	C20—H20C	0.9800
C1—C6	1.402 (4)	C21—H21A	0.9800
C2—C3	1.381 (4)	C21—H21B	0.9800
C2—H2A	0.9500	C21—H21C	0.9800
C3—C4	1.398 (4)	O3—C22	1.414 (10)
C3—H3	0.9500	O3—H3A	0.8400
C4—C5	1.367 (4)	C22—H22A	0.9800
C4—H4	0.9500	C22—H22B	0.9800
C5—C6	1.397 (4)	C22—H22C	0.9800
C5—H5	0.9500	O3'—C22'	1.324 (19)
C7—C8	1.459 (4)	O3'—H3'	0.8400
C8—C9	1.389 (3)	C22'—H22D	0.9800
C9—C10	1.383 (4)	C22'—H22E	0.9800
C9—H9	0.9500	C22'—H22F	0.9800
C10—C11	1.374 (4)		
			
C20—O1—H1	109.5	N5—C12—C11	122.3 (3)
C21—O2—H2	109.5	N5—C12—C13	114.4 (2)
C7—N1—C1	107.3 (2)	C11—C12—C13	123.4 (2)
C7—N1—H1N	126 (2)	N4—C13—N3	112.8 (2)
C1—N1—H1N	126 (2)	N4—C13—C12	126.1 (2)
C7—N2—C6	104.5 (2)	N3—C13—C12	121.0 (2)
C13—N3—C19	107.4 (2)	N4—C14—C15	130.3 (3)
C13—N3—H3N	128 (3)	N4—C14—C19	109.9 (2)
C19—N3—H3N	125 (3)	C15—C14—C19	119.8 (3)
C13—N4—C14	105.0 (2)	C16—C15—C14	117.8 (3)
C8—N5—C12	117.9 (2)	C16—C15—H15	121.1
N1—C1—C2	132.8 (3)	C14—C15—H15	121.1
N1—C1—C6	105.1 (2)	C15—C16—C17	121.4 (3)
C2—C1—C6	122.1 (3)	C15—C16—H16	119.3
C3—C2—C1	116.7 (3)	C17—C16—H16	119.3
C3—C2—H2A	121.6	C18—C17—C16	121.7 (3)
C1—C2—H2A	121.6	C18—C17—H17	119.1
C2—C3—C4	121.4 (3)	C16—C17—H17	119.1
C2—C3—H3	119.3	C17—C18—C19	116.9 (3)
C4—C3—H3	119.3	C17—C18—H18	121.6
C5—C4—C3	122.2 (3)	C19—C18—H18	121.6
C5—C4—H4	118.9	N3—C19—C18	132.6 (3)
C3—C4—H4	118.9	N3—C19—C14	104.9 (2)
C4—C5—C6	117.3 (3)	C18—C19—C14	122.4 (3)
C4—C5—H5	121.4	O1—C20—H20A	109.5
C6—C5—H5	121.4	O1—C20—H20B	109.5
C5—C6—N2	129.6 (3)	H20A—C20—H20B	109.5
C5—C6—C1	120.4 (3)	O1—C20—H20C	109.5
N2—C6—C1	110.1 (2)	H20A—C20—H20C	109.5
N2—C7—N1	113.1 (2)	H20B—C20—H20C	109.5
N2—C7—C8	126.1 (2)	O2—C21—H21A	109.5
N1—C7—C8	120.8 (2)	O2—C21—H21B	109.5
N5—C8—C9	123.3 (2)	H21A—C21—H21B	109.5
N5—C8—C7	114.4 (2)	O2—C21—H21C	109.5
C9—C8—C7	122.2 (2)	H21A—C21—H21C	109.5
C10—C9—C8	118.2 (3)	H21B—C21—H21C	109.5
C10—C9—H9	120.9	C22'—O3'—H3'	109.5
C8—C9—H9	120.9	O3'—C22'—H22D	109.5
C11—C10—C9	119.5 (2)	O3'—C22'—H22E	109.5
C11—C10—H10	120.3	H22D—C22'—H22E	109.5
C9—C10—H10	120.3	O3'—C22'—H22F	109.5
C10—C11—C12	118.8 (2)	H22D—C22'—H22F	109.5
C10—C11—H11	120.6	H22E—C22'—H22F	109.5
C12—C11—H11	120.6		
			
C7—N1—C1—C2	179.1 (3)	C9—C10—C11—C12	0.2 (4)
C7—N1—C1—C6	−0.4 (3)	C8—N5—C12—C11	−0.5 (3)
N1—C1—C2—C3	179.4 (3)	C8—N5—C12—C13	−179.5 (2)
C6—C1—C2—C3	−1.1 (4)	C10—C11—C12—N5	0.4 (4)
C1—C2—C3—C4	0.1 (4)	C10—C11—C12—C13	179.4 (2)
C2—C3—C4—C5	0.4 (4)	C14—N4—C13—N3	1.0 (3)
C3—C4—C5—C6	0.0 (4)	C14—N4—C13—C12	−178.6 (2)
C4—C5—C6—N2	179.5 (2)	C19—N3—C13—N4	−0.9 (3)
C4—C5—C6—C1	−1.0 (4)	C19—N3—C13—C12	178.7 (2)
C7—N2—C6—C5	178.7 (3)	N5—C12—C13—N4	179.9 (2)
C7—N2—C6—C1	−0.9 (3)	C11—C12—C13—N4	0.8 (4)
N1—C1—C6—C5	−178.8 (2)	N5—C12—C13—N3	0.3 (3)
C2—C1—C6—C5	1.6 (4)	C11—C12—C13—N3	−178.7 (2)
N1—C1—C6—N2	0.8 (3)	C13—N4—C14—C15	178.1 (3)
C2—C1—C6—N2	−178.8 (2)	C13—N4—C14—C19	−0.7 (3)
C6—N2—C7—N1	0.6 (3)	N4—C14—C15—C16	−178.7 (3)
C6—N2—C7—C8	179.5 (2)	C19—C14—C15—C16	0.1 (4)
C1—N1—C7—N2	−0.1 (3)	C14—C15—C16—C17	−0.2 (4)
C1—N1—C7—C8	−179.1 (2)	C15—C16—C17—C18	0.6 (5)
C12—N5—C8—C9	−0.1 (3)	C16—C17—C18—C19	−0.9 (4)
C12—N5—C8—C7	−179.9 (2)	C13—N3—C19—C18	−177.7 (3)
N2—C7—C8—N5	−178.7 (2)	C13—N3—C19—C14	0.4 (3)
N1—C7—C8—N5	0.1 (3)	C17—C18—C19—N3	178.6 (3)
N2—C7—C8—C9	1.5 (4)	C17—C18—C19—C14	0.8 (4)
N1—C7—C8—C9	−179.7 (2)	N4—C14—C19—N3	0.2 (3)
N5—C8—C9—C10	0.7 (4)	C15—C14—C19—N3	−178.7 (2)
C7—C8—C9—C10	−179.6 (2)	N4—C14—C19—C18	178.6 (2)
C8—C9—C10—C11	−0.7 (4)	C15—C14—C19—C18	−0.4 (4)

### Hydrogen-bond geometry (Å, °)

**Table:**

*D*—H···*A*	*D*—H	H···*A*	*D*···*A*	*D*—H···*A*
O1—H1···O2^i^	0.84	1.83	2.670 (3)	176
O2—H2···N4	0.84	1.91	2.741 (3)	168
N1—H1N···O1	0.87 (1)	2.07 (1)	2.927 (3)	171 (3)
N3—H3N···O1	0.86 (1)	2.07 (1)	2.925 (3)	171 (4)

Symmetry codes: (i) −*x*+1, −*y*+1, −*z*+1.
